# Superiority of NBI endoscopy to PET/CT scan in detecting esophageal cancer among head and neck cancer patients: a retrospective cohort analysis

**DOI:** 10.1186/s12885-020-6558-4

**Published:** 2020-01-29

**Authors:** Hsuan-An Su, Shun-Wen Hsiao, Yu-Chun Hsu, Lien-Yen Wang, Hsu-Heng Yen

**Affiliations:** 1 Department of Medical Education, Kaohsiung Chang Gung Memorial Hospital, Kaohsiung, Taiwan; 2 Endoscopy Center, Changhua Christian Hospital, 135 Nanhsiao Street, Changhua, Taiwan; 3 Department of Nuclear Medicine, Changhua Christian Hospital, Changhua, Taiwan; 4 Institute of Medicine, Chung Shan Medical University, Taichung, Taiwan; 5 General Education Center, Chienkuo Technology University, Changhua, Taiwan

**Keywords:** PET/CT scan, Narrow-band imaging, Endoscopy, Head and neck cancer, Screening, Second primary cancer, Squamous cell carcinoma, Esophageal cancer

## Abstract

**Background:**

Second primary cancer of the esophagus is frequent in head and neck patients, especially in high-risk populations, and has a great impact on the prognosis. Although Positron emission tomography (PET)/computed tomography (CT) scan is commonly conducted in head and neck patients, its ability to detect early esophageal cancer is limited. Narrow-band imaging endoscopy is an accurate and convenient technique for esophageal examination. We aimed to compare PET/CT scan and narrow-band imaging endoscopy for the detection of esophageal cancer in head and neck cancer patients.

**Methods:**

From November 2015 to November 2018, all head and neck cancer patients who underwent both PET/CT scan and narrow-band imaging endoscopy at Changhua Christian Hospital were retrospectively enrolled. Descriptive statistics, receiver operating characteristic curve analysis, logistic regression analysis, independent Student’s t-test, and Kaplan–Meier survival analysis were conducted with MedCalc Statistical Software.

**Results:**

A total of 147 subjects were included in the analysis; suspicious esophageal lesions were identified by PET/CT scan in 8 (5.44%) and by narrow-band imaging in 35 (23.81%). The final pathologic diagnoses were esophageal squamous cell carcinoma in 10 and high-grade dysplasia in 5. The respective sensitivity, specificity, and area under the curve for detecting suspicious esophageal lesions were 33.33, 97.73%, and 0.655 for PET/CT scan, and 100.0, 84.85%, and 0.924 for narrow-band imaging endoscopy. Hypopharyngeal or laryngeal location of the primary head and neck cancer was the only risk factor for developing second primary esophageal cancer.

**Conclusions:**

PET/CT scan was inferior to narrow-band imaging endoscopy in detecting second primary esophageal cancer in head and neck cancer patients. In addition to PET/CT scan, narrow-band imaging endoscopy should be considered in head and neck patients at high risk for developing second primary esophageal cancer.

## Background

Head and neck cancers (HNC) account for approximately 3.7% of all cancers and 2.4% of all cancer-related deaths in both sexes in the United States [1]. In Taiwan, HNC accounts for approximately 10% of all cancers, with a 7.3-fold higher incidence in men than in women, and about 8.2% of all cancer-related death in both sexes [2].

Second primary cancers (SPC) in the respiratory and gastrointestinal tracts, such as the lungs and the esophagus, had been frequent in patients with HNC. SPC of the esophagus was shown to develop in about 7.4 to 51.5% of HNC patients [3–5], with a relative risk of 23 among oral cancer patients [6] and a standardized 5.9-fold higher risk in HNC patients, compared with the baseline incidence [7]. The existence of a second primary esophageal squamous cell carcinoma (ESCC) had been reported as an independent detrimental prognostic factor in HNC patients and significantly reduced overall survival, with hazard ratios ranging from 1.53 to 2.75 [8–14]. Early detection of SPC of the esophagus, endoscopic resection, and implementation of pretreatment endoscopic screening policy had been shown to significantly improve the survival rates of HNC patients, especially among Asians [9, 12, 13, 15].

Endoscopy with narrow-band imaging (NBI) is one of the virtual chromoendoscopic techniques that enhance blue and green light to highlight abnormal neoplastic vasculatures, based on the different optic absorbability values of hemoglobin at certain wavelengths [16]. NBI endoscopy had been shown to be superior to Lugol chromoendoscopy in detecting ESCC [17]. In a systematic review and meta-analysis, the sensitivity and specificity of NBI endoscopy for the diagnosis of SPC of the esophagus were 97 and 94%, respectively [18]. Positron emission tomography (PET)/computed tomography (CT) scan had been clinically useful for the staging, detecting distant metastases or SPC, and tumor surveillance of HNC [19], but it was reported to be unsuitable for screening and detecting early or superficial esophageal cancer [20]. Although routine endoscopic examination for SPC of the esophagus in HNC patients was advocated by studies on Asian populations [10, 21–25], it was not recommended by researchers from Western countries, except for patients with high-risk factors for SPC of the esophagus, such as tobacco or alcoholic exposure and specific sites of the primary HNC [18, 26–35].

Some studies have investigated the efficacy of PET/CT scan and compared the different endoscopic modalities in detecting esophageal SPCs, but a direct comparison between PET/CT scan and NBI endoscopy in newly diagnosed head and neck squamous cell carcinoma (HNSCC) patients had not been conducted. In the present retrospective study, we aimed to evaluate the role of PET/CT scan, in comparison with NBI endoscopy, in detecting SPC of the esophagus in newly diagnosed HNSCC patients.

## Methods

### Study design and data extraction

The implementation of a universal NBI endoscopy screening program for patients with HNC had been initiated at the Endoscopy Center of Changhua Christian Hospital in Taiwan since November 2015. All patients were referred to gastroenterologists for NBI endoscopy, as part of the disease staging work-up. All patients provided written informed consent before endoscopic examination. The present retrospective study enrolled newly diagnosed HNC patients who underwent initial stage work-up by both PET/CT scan and NBI endoscopy and who received the complete treatment course at our hospital from November 2015 to November 2018. Patients who had been previously diagnosed with other malignancies, those did not complete the treatment course at our hospital, or those with incomplete follow-up data were excluded. This study was approved by the institutional review board of Changhua Christian Hospital (approval number: CCH IRB 180702).

### Protocol of NBI endoscopic examination

All patients underwent endoscopic examination with an NBI system (Evis Lucera CLV-260NBI endoscopy, Olympus Medical Systems Corp., Tokyo, Japan). All endoscopic examinations and endoscopic submucosal dissection of ESCC were performed by four experienced endoscopists who had performed more than 500 endoscopy cases annually. After an overnight fast, the patients were asked to swallow a dose of simethicone solution, followed by local pharyngeal anesthesia with 10% xylocaine spray. First, the esophagus was examined from the esophageal inlet to the esophagogastric junction by conventional white-light endoscopy, followed by repeat evaluation from the distal to the proximal under NBI. A suspicious esophageal lesion was defined as a brownish discoloration of the mucosa, with abnormal vascular patterns under the NBI magnifying endoscopy system. Endoscopic biopsy was done for all suspicious lesions.

### Protocol for ^18^F-FDG PET/CT scan

Blood glucose levels were measured to check for potential hyperglycemia and patients had to stay sober for at least six hours before the intravenous injection of ^18^F-FDG at 0.1–0.2 mCi/kg. After ^18^F-FDG administration, whole-body PET/CT scan was performed at 60 min and delayed regional imaging was performed at 120 min. Non-contrast, low-dose CT scan from the skull vertex to the mid-thigh was conducted for attenuation correction and anatomical localization. All ^18^F-FDG PET/CT images were visually interpreted by an experienced nuclear medicine physicians, and an SUV_max_ value ≧3.5 was highly considered as a positive PET/CT result [36, 37].

### Statistical analysis

The extracted data were organized using Microsoft Excel software and were analyzed by MedCalc Statistical Software version 18.11 (MedCalc Software bvba, Ostend, Belgium; https://www.medcalc.org; 2018). Descriptive statistics, receiver operating characteristic curve (ROC), logistic regression analysis, independent Student’s t-test, and Kaplan–Meier survival analysis were used to elaborate the demographic and crude estimates, to evaluate the diagnostic abilities of the examinations, to analyze the risk factors for the diseases of interest, to compare continuous variables between two groups, and to illustrate the survival outcomes of patients under specific circumstances, respectively. Statistical significance was considered for *p <* 0.05.

## Results

**Fig. 1:**
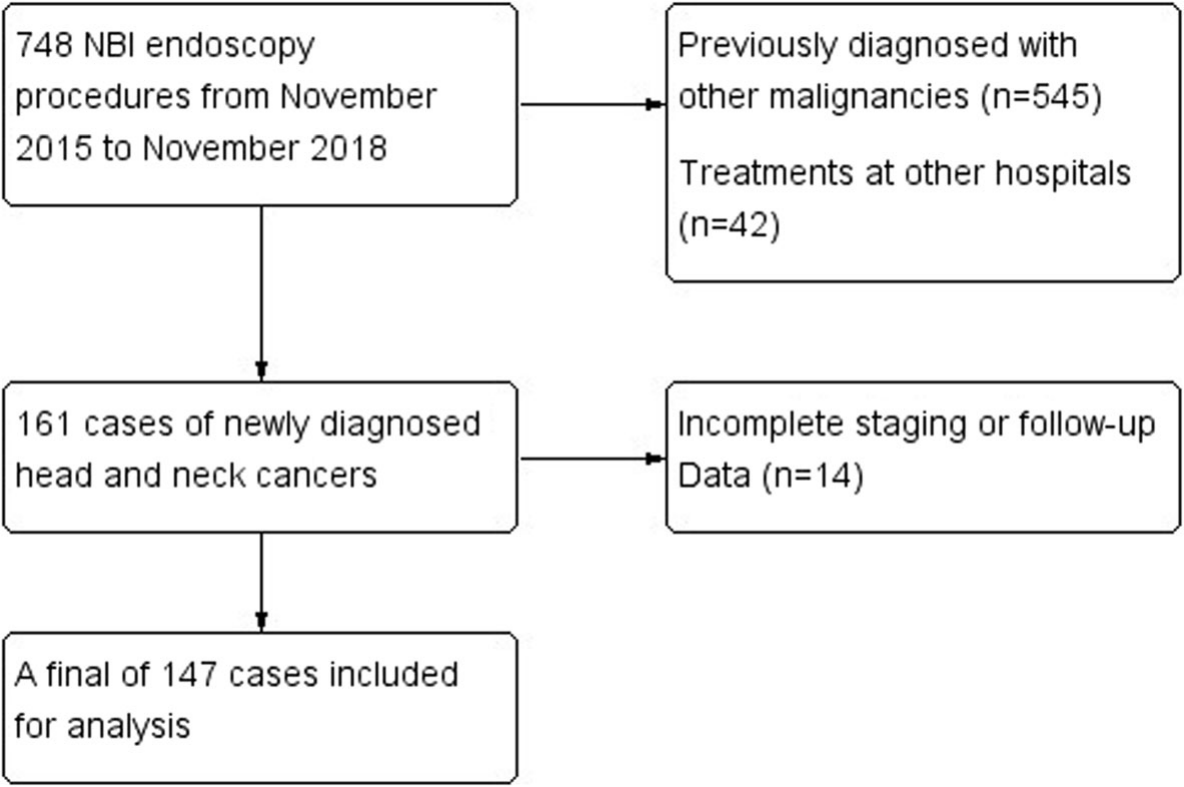
Flowchart for study design

Initially, a total of 748 records of NBI procedures were identified from the database of the Endoscopy Center of Changhua Christian Hospital from November 2015 to November 2018. After excluding subjects based on our defined criteria, a total of 147 subjects were finally enrolled in the analysis (Fig. 1).

As shown in Table 1, the study population comprised 143 (97.3%) men and had a mean ± standard deviation age of 54.17 ± 10.87 years (range, 19–84 years). Habitual consumption of alcohol, betel-nuts, and cigarettes were prevalent in our subjects, with percentages of 74.83, 80.95, and 89.16%, respectively. The locations of the primary HNC were the nasopharynx (*N* = 3, 2.04%); oral cavity (*N* = 98, 66.67%); oropharynx (*N* = 17, 11.56%); hypopharynx (*N* = 28, 19.05%); and larynx (N = 1, 0.68%). Only two patients had metastatic lesions of the primary HNC to the lung and liver, respectively. According to the American Joint Committee on Cancer Seventh Edition TNM Staging System, 20 (13.61%) subjects were categorized as stage I; 22 (14.97%) were stage II; 10 (6.80%) were stage III; 91 (61.90%) were stage IV; and 4 (2.72%) were of unknown/inapplicable stage. Suspicious esophageal lesions were found by PET/CT scan in 8 (5.44%) and by NBI endoscopy in 35 (23.81%). Of 147 HNC patients, 10.2% had ESCC (*N* = 10) and severe or high-grade dysplasia (*N* = 5) on pathologic examination of the endoscopic biopsy samples. In addition, gastric ulcer, duodenal ulcer, and positive Campylobacter-like organism test were identified in 39/144 (27.08%), 32/145 (22.07%), and 36/134 (26.87%) of the patients, respectively.

**Table 1:** Basic characteristics of the subjects

Characteristics	Values
N	147
Sex (M/F)	143/4 (97.3%/2.7%)
Age (mean, SD)	54.17 ± 10.87 (19–84)
Substance use	
Alcohol consumption	110 (74.83%)
Betel-nut consumption	119 (80.95%)
Cigarette consumption	131 (89.16%)
HNC tumor location	
Nasopharynx	3 (2.04%)
Oral cavity	98 (66.67%)
Oropharynx	17 (11.56%)
Hypopharynx	28 (19.05%)
Larynx	1 (0.68%)
Metastatic lesions	
No	143 (97.28%
Yes	2 (1.36%)
Unknown	2 (1.36%)
Seventh AJCC Staging of the HNSCC	
Stage I	20 (13.61%)
Stage II	22 (14.97%)
Stage III	10 (6.80%)
Stage IVA	65 (44.22%)
Stage IVB	24 (16.33%)
Stage IVC	2 (1.36%)
Unknown	4 (2.72%)
PET/CT scan findings of the esophagus	
Positive	8 (5.44%)
Negative	139 (94.56%)
Endoscopic findings	
Suspicious esophageal lesion	35 (23.81%)
Gastric ulcer	39 (27.08%)
Duodenal ulcer	32 (22.07%)
Pathologic findings in the esophagus	
Squamous cell carcinoma	10 (6.80%)
High-grade dysplasia	5 (3.40%)
CLO test positivity	36 (26.87%)

*AJCC* American Joint Committee on Cancer, *SD* standard deviation, *HNSCC* head and neck squamous cell carcinoma, *CLO* Campylobacter-like organism

**Fig. 2:**
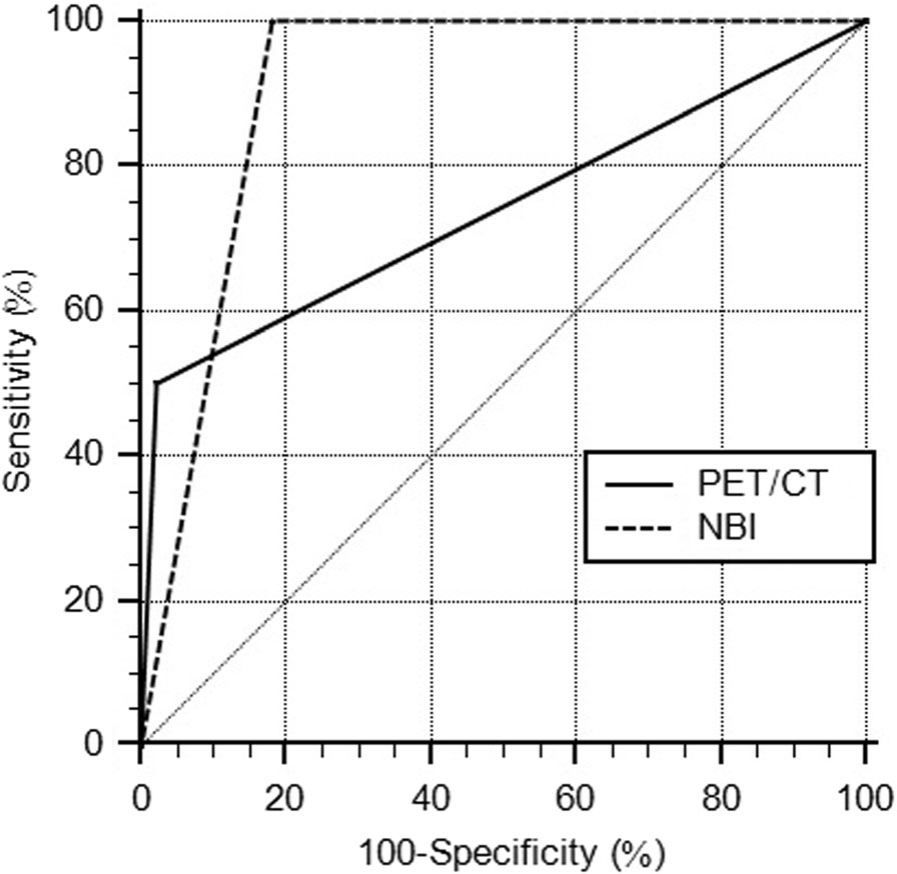
The ROC curves of PET/CT scan and NBI endoscopy on diagnosing ESCC The AUC is significantly larger for NBI endoscopy than for PET/CT scan (*p =* 0.046).

**Fig. 3:**
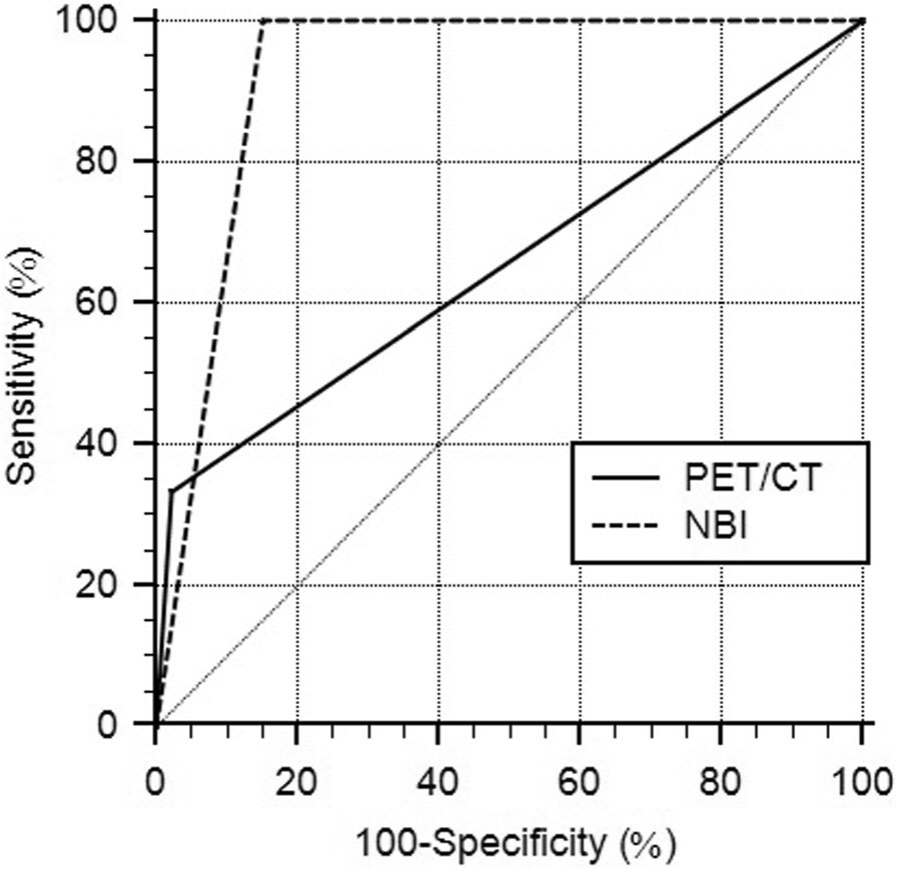
The ROC curves of PET/CT scan and NBI endoscopy on diagnosing suspicious esophageal lesions The AUC is significantly larger for NBI endoscopy than for PET/CT scan (*p* < 0.001)

On ROC analysis (Table 2), the respective sensitivities and specificities for ESCC detection were 50 and 97.81% for PET/CT scan and 100 and 81.75% for NBI endoscopy; the area under the curve (AUC) values were 0.739 [95% confidence interval (CI), 0.660–0.808; *p =* 0.004] for PET/CT scan and 0.909 (95% CI, 0.850–0.950; *p* < 0.001) for NBI endoscopy (Fig. 2). In addition, the respective sensitivities and specificities for detecting suspicious esophageal lesions, including ESCC and high-grade dysplasia, were 33.33 and 97.73% for PET/CT scan and 100 and 84.85% for NBI endoscopy; the AUC values were 0.655 (95% CI, 0.573–0.732; *p* = 0.014) for PET/CT scan and 0.924 (95% CI, 0.869–0.961; *p* < 0.001) for NBI endoscopy (Fig. 3). The ROCs of PET/CT scan and NBI endoscopy were significantly different for detecting ESCC (*p* = 0.046) and for detecting suspicious esophageal lesions (*p* < 0.001).

**Table 2:** Comparison between PET/CT scan and NBI endoscopy

	Sensitivity	Specificity	AUC
ESCC			
PET/CT scan	50.00%	97.81%	0.739 (0.660–0.808)
NBI	100.0%	81.75%	0.909 (0.850–0.950)
Suspicious esophageal lesions^a^			
PET/CT scan	33.33%	97.73%	0.655 (0.573–0.732)
NBI	100.0%	84.85%	0.924 (0.869–0.961)

*AUC* area under the curve, *ESCC* esophageal squamous cell carcinoma

^a^include both ESCC and high-grade dysplasia

**Fig. 4:**
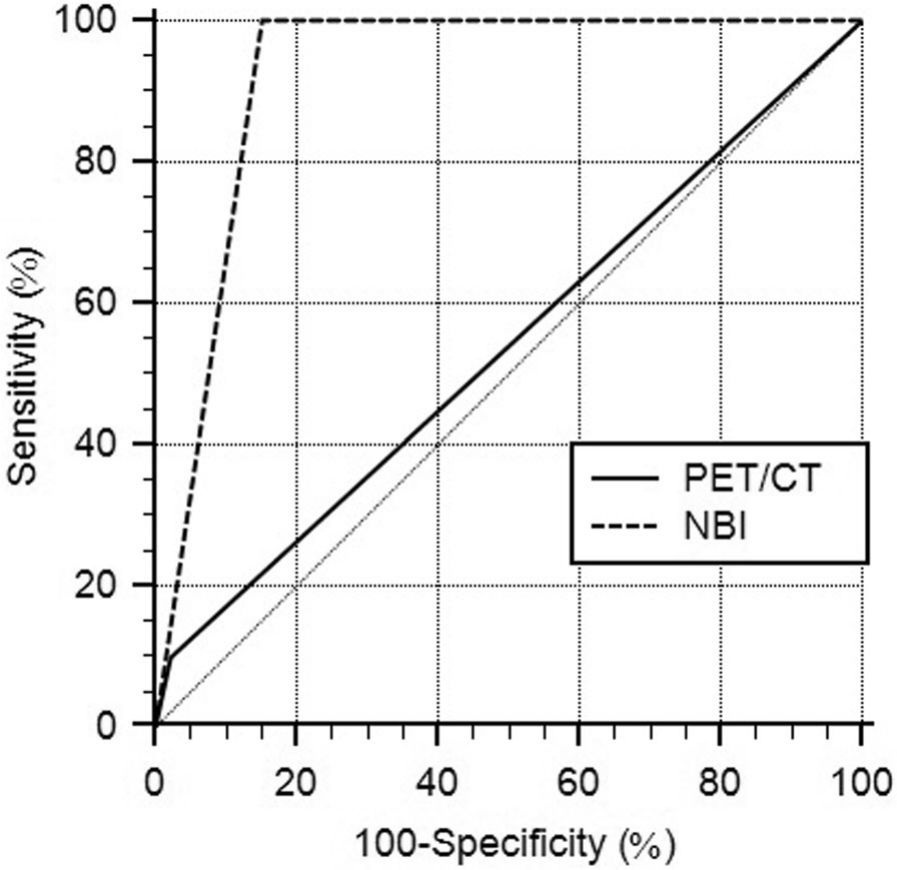
PET/CT scan and NBI endoscopy’s ROC curves on diagnosing superficial esophageal lesions (Tis and T1) The AUC of NBI endoscopy is significantly larger than that of PET/CT scan (*p* < 0.001)

**Fig. 5:**
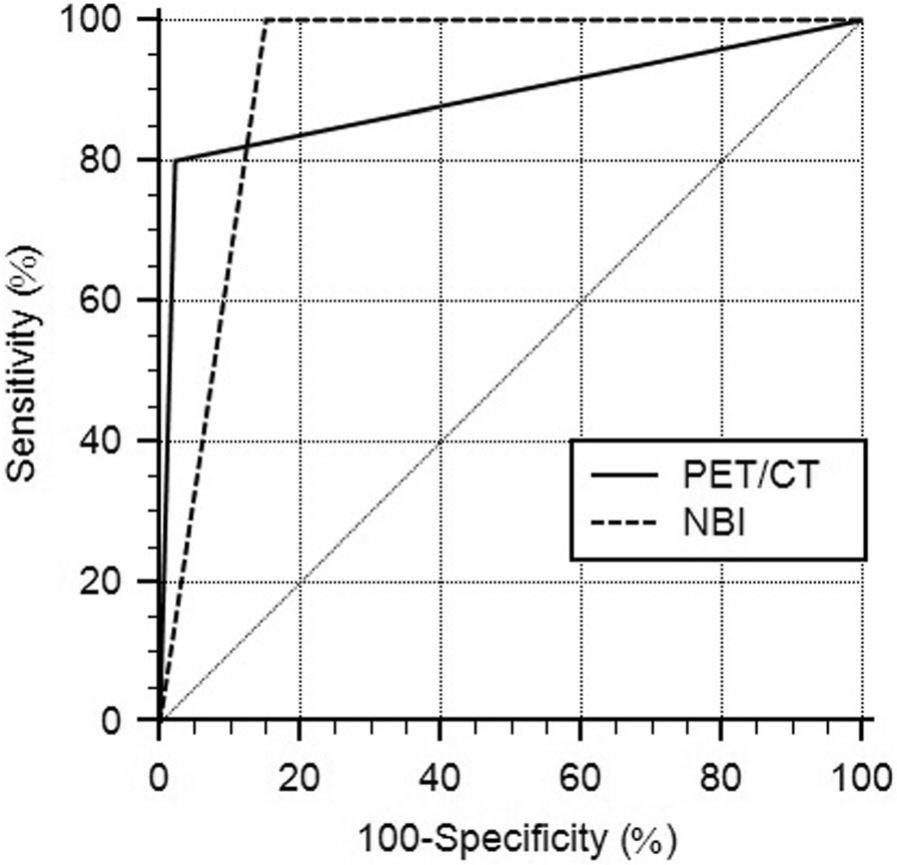
PET/CT scan and NBI endoscopy’s ROC curves on diagnosing deep esophageal lesions (T2 and T3)

The distribution of the detected esophageal lesions, according to the T classification, on PET/CT scan and NBI endoscopy is listed in Table 3. The AUCs for detecting superficial lesions (Tis and T1) were 0.539 for PET/CT scan and 0.924 for NBI endoscopy (Fig. 4; *p* < 0.001). On the other hand, the AUCs for detecting deep lesions (T2 and T3) were 0.889 for PET/CT scan and 0.924 for NBI endoscopy (Fig. 5; *p =* 0.725).

**Table 3:** Esophageal lesions’ distribution, according to the clinical T classification, and the diagnostic abilities of PET/CT and NBI

T classification	ESCC or high-grade dysplasia	NBI-positive lesions	NBI-negative lesions	PET/CT-positive lesions	PET/CT-negative lesions
Negative	132	20	112	3	129
Tis	5	5	0	0	5
T1	5	5	0	1	4
T2	3	3	0	2	1
T3	2	2	0	2	0
T4	0	0	0	0	0

*ESCC* esophageal squamous cell carcinoma

The AUC of NBI endoscopy is not significantly different from that of PET/CT scan (*p =* 0.725).

The risk factors for SPC of the esophagus in the HNC patients were investigated by stepwise logistic regression. Among the variables, including age, sex, high-risk HNC location, and advanced stage of HNC, only high-risk HNC location was predictive of the risk for development of SPC of the esophagus, with an odds ratio (OR) of 4.7 (95% CI, 1.26–17.55; *p =* 0.025). Likewise, the OR of high-risk HNC location for the development of suspicious esophageal lesions was 4.38 (95% CI, 1.44–13.31; *p =* 0.012).

**Fig. 6:**
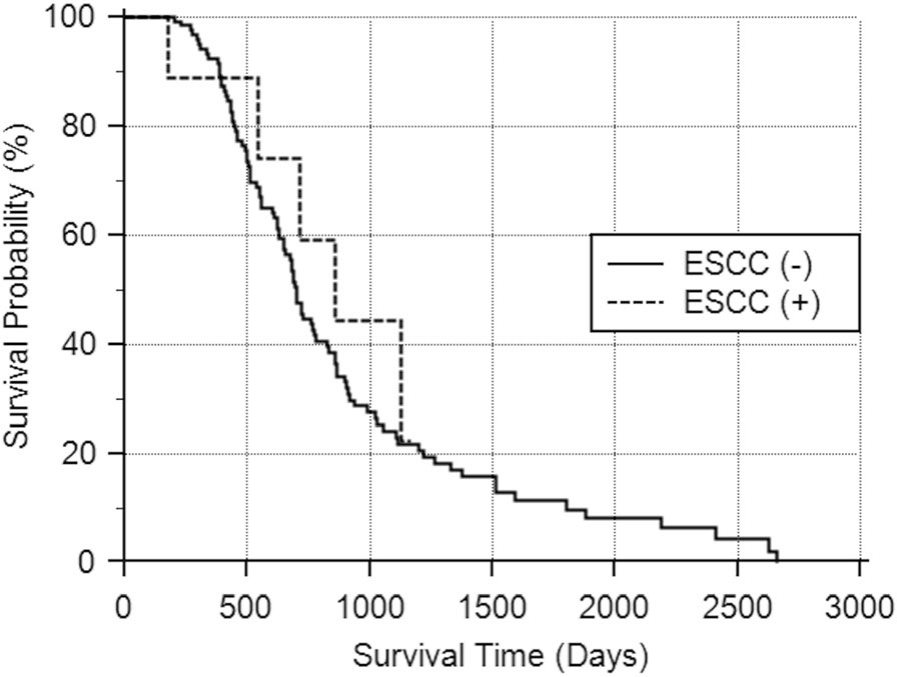
The overall survival curves of HNC patients, according to the presence of ESCC. The overall survival rates are similar between patients with ESCC and those without ESCC (*p =* 0.5934)

The follow-up period ranged from 1 to 88.6 months, with a mean of 23.2 months. As shown in Fig. 6, the two-year overall survival rates of HNC patients were 44.56% for those with ESCC and 57.70% for those without ESCC (*p =* 0.5934).

Of the four endoscopists in the present study, two were high-volume endoscopists (115 cases) and the other two were relatively low-volume endoscopists (32 cases). Based on independent Student’s t-test, there were no significant differences between the two groups of endoscopists in detecting ESCC (*p =* 0.8304) and suspicious esophageal lesions (*p =* 0.4096).

## Discussion

In the present study, we aimed to compare the diagnostic abilities between NBI endoscopy and PET/CT scan for detecting SPC of the esophagus in HNC patients. This was the first report that directly compared the use of the two modalities for HNC patients. Our major findings revealed that compared with PET/CT scan, NBI endoscopy was superior and had 100% sensitivity for detecting ESCC and suspicious esophageal lesions, although its specificity was slightly lower. The higher AUC of NBI endoscopy, compared with that of PET/CT scan, implied the better performance of the former in detecting SPC of the esophagus in HNC patients.

A systematic review and meta-analysis reported similar results for NBI endoscopy, with 97% sensitivity and 94% specificity for detecting esophageal cancer in HNC patients [18]. In another study on a population that was not limited to HNC patients, NBI endoscopy yielded satisfactory sensitivity and specificity for diagnosing ESCC (88 and 88%, respectively, on per-patient analysis; 94 and 65%, respectively, on per-lesion analysis) [17]. The diagnostic performances of the different image-enhanced endoscopic (IEE) modalities had been compared in some studies. In a systematic review, NBI endoscopy performed better than Lugol chromoendoscopy in detecting SPC of the esophagus in HNC patients [18]. Another systematic review concluded that NBI endoscopy was superior to Lugol chromoendoscopy in the overall detection of ESCC [17]. A relatively recent analysis reported equivalent performances of the two in detecting early ESCC [38], but the use of NBI was more convenient and can prevent the potential harms (i.e., allergy) of iodine dye, compared with the use of Lugol chromoendoscopy [17].

Because the performance of endoscopy is operator-dependent and requires clinical experience and skills, another advantage of NBI endoscopy over white-light endoscopy is that it can reduce operator dependence. The insignificant results by independent Student’s t-test between high-volume and low-volume endoscopists suggested that the NBI endoscopy measurements were in acceptable agreement between the two groups of endoscopists. Although evaluation of interrater agreement could have been better analyzed by Cohen’s kappa coefficient, our dataset lacked repeated measurements, which made kappa analysis not feasible. Further analysis of interrater agreements on NBI techniques is required.

Our results indicated imperfect performance of PET/CT scan in detecting SPC of the esophagus. The limited spatial resolution of PET/CT scan was likely the primary cause of its inability to visualize early ESCC [39]. Moreover, ^18^F-FDG uptake had been shown to be positively correlated with the depth of ESCC [40, 41]; therefore, superficial SCC was less likely to be detected on PET/CT scan. Our results demonstrated that esophageal tumor depth or T classification was associated with the diagnostic abilities of PET/CT and NBI endoscopy. Likewise, other studies reported that PET/CT scan was suitable for deeper lesions, whereas endoscopy performed well in identifying both deep and superficial lesions [5, 41]. Furthermore, false-positive PET/CT scan may be seen in numerous clinical situations, including gastroesophageal reflux disease, other esophageal inflammatory conditions, benign neoplasms, and increased uptake in normal tissues, such as the muscles [20, 39]. Despite its inferior performance to NBI endoscopy in detecting SPC of the esophagus, PET/CT scan remains indispensable for HNC patients. The various uses of PET/CT scan had been for disease staging, decision on treatment strategy, evaluation of prognosis, follow-up after treatment, and, most importantly, identification of unknown primary or metastatic neoplasms [19].

As previously mentioned, IEE had been advocated for routine screening for SPC of the esophagus in patients with HNSCC/HNC; however, most of the studies, including a systematic review and meta-analysis, were from far eastern countries [10, 18, 21–23]. In another systematic review, in which 80% of the included studies were from far eastern countries, IEE was strongly recommended for screening SPC in Asian populations; however, the role of IEE for screening remained unclear in Western populations [24]. Most of the published studies and clinical guidelines supported the role of routine IEE in high-risk populations only; among these publications, the descriptions of a high-risk population were variable and include the degree of tobacco and alcohol use; specific locations of the primary HNC, most were in the hypopharynx and larynx, as shown in our results; presence of esophageal symptoms; and ethnicity of the patients [28–32, 34, 35, 42].

Because the survival curves of our HNC patients did not differ according to the presence of ESCC, the benefit screening for SPC of the esophagus in HNC patients was not supported by our analyses. Nevertheless, the importance of screening for SPC of the esophagus cannot be overlooked. Early diagnosis of the SPC of the esophagus had been suggested [10, 14, 22, 23, 25, 43] or proven [9, 13, 15, 25] to have significant benefits on the prognosis and survival rates of patients. In addition, the treatment strategy was reported to vary according to the presence of ESCC [18, 44]; about 15.5% of HNC cases had their treatment strategies modified after identification of SPC of the esophagus by endoscopy [45]. Because of the ability of endoscopy to identify superficial lesions (Tis and T1), such as SPC of the esophagus, which are less likely to be discovered on PET/CT scan, surgical procedures for the esophageal lesions might need to be replanned or radiotherapy might need to be modified to cover the thoracic region in patients who would require concurrent chemoradiotherapy. In our analysis, all 10 patients diagnosed as ESCC were further treated by surgery (*N* = 1), endoscopic submucosal dissection (*N* = 3), and concurrent chemoradiotherapy (*N* = 6).

Because of the high incidence of SPC of the esophagus with primary HNC in Asian populations, the improvements in prognosis by early detection of SPC of the esophagus, and the dependence of the treatment strategy on the existence of an SPC, we propose, once again, the implementation of routine IEE examination, especially with NBI endoscopy, in all newly diagnosed HNC patients in Asian populations. ESCC has a high incidence in Asian populations, whereas esophageal adenocarcinoma accounts for most of the esophageal cancer cases in the Western countries [46]. Therefore, it would be reasonable to perform screening for SPC of the esophagus only for high-risk populations. In Taiwan, the role of endoscopy remains to be optional, according to the clinical guidelines for HNC published by the Taiwan Head and Neck Society in 2016 [47]. Since the cost per life-year of treatment for esophageal cancer is one of the highest expenditures among cancer therapies in Taiwan [48], early identification by endoscopic screening should be highly encouraged, in order to save the financial budget of the National Health Insurance in a more cost-effective way.

A systematic review reported that the global prevalence of peptic ulcer disease (PUD) was approximately 0.12 to 4.7% [49], and the prevalence of asymptomatic PUD in Taiwan was 9.4% [50]. However, in the present study, we found a higher prevalence of PUD of up to 27.08%. HNC and PUD share some common risk factors, such as smoking and betel nut chewing [50]. Furthermore, the primary care physician visits, specialist referrals, stress, and depression in HNC patients [51] likely predisposed these patients to have the other risk factors for PUD and may partially explain the high prevalence of PUD in our population. Although the risk factors for the development of PUD in HNC patients were not investigated in the present study, we would like to emphasize the high risk for PUD in HNC patients. In particular, physicians should be aware of the possibility of peptic ulcer bleeding after major therapeutic events, such as surgery, and should consider the use of proton-pump inhibitors, whenever clinically indicated.

We acknowledged some limitations of the present study. First, the retrospective design made selection and reporting biases inevitable to a certain degree, and the limited sample size could have resulted to sampling bias. Second, endoscopy was performed by four examiners; there may have been inconsistencies among the operators, and interrater analysis was not feasible in the current dataset. Nevertheless, we believed that these limitations had little influence on our major objective of comparing the diagnostic ability between PET/CT scan and NBI endoscopy.

## Conclusions

Based on the high incidence and great prognostic impact of SPC of the esophagus in HNC patients, PET/CT scan alone may be insufficient for the staging work-up of HNC, because it had considerable limitations in detecting early esophageal cancer in high-risk populations. The performance of NBI endoscopy was superior to PET/CT scan in detecting SPC of the esophagus in HNC patients.

## Data Availability

The datasets generated and/or analyzed during the current study are not publicly available, due to the privacy of the enrolled subjects, but these may be requested from the corresponding author, upon reasonable request.

## Abbreviations

^18^F-FDG: ^18^F-labeled fluoro-2-deoxyglucose
ESCC: Esophageal squamous cell carcinoma
HNC: Head and neck cancer
HNSCC: Head and neck squamous cell carcinoma
IEE: Image-enhanced endoscopy
NBI: Narrow-band imaging
PET: Positron emission tomography
PUD: Peptic ulcer disease
ROC: Receiver operating characteristic
SCC: Squamous cell carcinoma
SPC: Second primary cancer
